# Prostate Cancer Treatment with Pencil Beam Proton Therapy Using Rectal Spacers sans Endorectal Balloons

**DOI:** 10.14338/IJPT-21-00039

**Published:** 2022-04-06

**Authors:** Matthew Forsthoefel, Ryan Hankins, Elizabeth Ballew, Cara Frame, David DeBlois, Dalong Pang, Pranay Krishnan, Keith Unger, Keith Kowalczyk, John Lynch, Anatoly Dritschilo, Sean P. Collins, Jonathan W. Lischalk

**Affiliations:** 1 Department of Radiation Medicine, Georgetown University Hospital, Washington, DC, USA.; 2 Department of Urology, Georgetown University Hospital, Washington, DC, USA.; 3 Department of Radiology, Georgetown University Hospital, Washington, DC, USA.; 4 Department of Radiation Oncology, Perlmutter Cancer Center at New York University Langone Hospital – Long Island, New York, NY, USA

**Keywords:** prostate cancer, radiation therapy, proton therapy, rectal spacer, endorectal balloon

## Abstract

**Purpose:**

Proton beam radiotherapy (PBT) has been used for the definitive treatment of localized prostate cancer with low rates of high-grade toxicity and excellent patient-reported quality-of-life metrics. Technological advances such as pencil beam scanning (PBS), Monte Carlo dose calculations, and polyethylene glycol gel rectal spacers have optimized prostate proton therapy. Here, we report the early clinical outcomes of patients treated for localized prostate cancer using modern PBS–PBT with hydrogel rectal spacing and fiducial tracking without the use of endorectal balloons.

**Materials and Methods:**

This is a single institutional review of consecutive patients treated with histologically confirmed localized prostate cancer. Prior to treatment, all patients underwent placement of fiducials into the prostate and insertion of a hydrogel rectal spacer. Patients were typically given a prescription dose of 7920 cGy at 180 cGy per fraction using a Monte Carlo dose calculation algorithm. Acute and late toxicity were evaluated using the Common Terminology Criteria for Adverse Events (CTCAE), version 5. Biochemical failure was defined using the Phoenix definition.

**Results:**

From July 2018 to April 2020, 33 patients were treated (median age, 75 years). No severe acute toxicities were observed. The most common acute toxicity was urinary frequency. With a median follow-up of 18 months, there were no high-grade genitourinary late toxicities; however, one grade 3 gastrointestinal toxicity was observed. Late erectile dysfunction was common. One treatment failure was observed at 21 months in a patient treated for high-risk prostate cancer.

**Conclusion:**

Early clinical outcomes of patients treated with PBS–PBT using Monte Carlo–based planning, fiducial placement, and rectal spacers sans endorectal balloons demonstrate minimal treatment-related toxicity with good oncologic outcomes. Rectal spacer stabilization without the use of endorectal balloons is feasible for the use of PBS–PBT.

## Introduction

Proton beam radiotherapy (PBT) was used for the definitive treatment of localized prostate cancer as early as the 1970s, when it was employed as a radiation boost [1]. Randomized evidence in the 1990s, with the publication of PROG 95-02, established PBT as an excellent means of dose escalation to improve biochemical progression-free survival (bPFS) [2]. For many decades, only a handful of proton facilities existed in the United States, which presented a barrier to the radiation oncology community for conducting phase III trials exploring the comparative effectiveness of PBT versus x-ray–based therapies. Single institutions, including the University of Florida, have published their long-term experience treating localized prostate cancer with PBT and have reported excellent rates of bPFS, low rates of high-grade toxicity, and excellent patient-reported quality of life [3]. In the modern era, PBT has become more ubiquitous, prompting the development of randomized trials such as the PARTIQoL, which aims to investigate the relative effectiveness of PBT versus standard x-ray therapy (NCT01617161) [4].

The physical dose superiority achieved with PBT underpins the rationale for its implementation in a variety of cancer sites, including genitourinary (GU) malignancies. This physical dose superiority has been further augmented by advances in the proton treatment planning (e.g., Monte Carlo optimization), technical delivery of PBT, and image guidance. Advancements in the delivery of PBT include the increased use of pencil beam scanning (PBS) technology, which allows for a more conformal dose distribution to be achieved, particularly along the proximal edge of the target compared with passive scatter PBT [5]. Image-guided radiotherapy (IGRT) enhanced by the placement of fiducial markers has allowed for radiation margin reduction to decrease surrounding normal tissue exposure to high-dose radiation. Furthermore, recent application of hydrogel rectal spacers has permitted creation of artificial geometric spacing between the prostate and rectum and ultimately optimizes dosimetry and minimizes radiation-related toxicity [6–12]. The use of endorectal balloons is commonplace in the delivery of PBT in an effort to stabilize pelvic anatomy, though there is little data as of yet to determine if rectal spacers alone offer stabilization and toxicity mitigation when combined with PBS–PBT.

We report the early clinical outcomes of patients treated for localized prostate cancer using modern PBS–PBT and Monte Carlo planning with hydrogel rectal spacing, sans endorectal balloons, and fiducial tracking at one of the first single-room PBS centers in the United States.

## Materials and Methods

### Patient Eligibility

This single institutional review of patients treated with localized prostate cancer was approved by the local Institutional Review Board. Men with histologically confirmed localized prostate cancer were eligible for analysis. All patients were evaluated by a multidisciplinary GU oncologic team, which included radiation oncology and urology. The AJCC 8th edition was used for staging, and patients were stratified into low-, intermediate-, and high-risk groups per the National Comprehensive Cancer Network risk classification [13, 14]. All patients underwent urological evaluation including prostate-specific antigen (PSA) levels, pretreatment biopsy, and staging imaging, commonly including computed tomography (CT) of the pelvis and bone scan, to confirm localized disease. Patients who received androgen-deprivation therapy (ADT) were classified into short-term administration if receiving up to 6 months of testosterone suppression and long-term administration if receiving over 6 months of testosterone suppression. Prostate volume was not used to determine candidacy for proton therapy but was taken into account for the duration of neoadjuvant ADT used, if any. All patients underwent implantation of fiducial markers into the prostate (Visicoil, IZI Medical Products, Owings Mill, Maryland) and placement of a hydrogel rectal spacer (Augmenix Inc, Bedford, Massachusetts) between the rectum and prostate by the Department of Urology. Hydrogel spacers were used in lieu of endorectal balloons for all patients in an effort to reduce radiation exposure to the rectum, mitigate toxicity rates, and improve reproducibility for proton therapy treatment via pelvic stabilization [7–12, 15]. Posterior extracapsular extension was considered exclusionary for the placement of rectal spacers. Patients with implanted cardiac devices were excluded from eligibility for PBT as an institutional standard.

### Simulation and Contouring

Patients underwent CT-based radiation treatment planning simulation (GE LightSpeed RT16, General Electric, Chicago, Illinois) with accompanied prostate magnetic resonance imaging (MRI). Patients were counseled to have a comfortably full bladder and empty rectum prior to simulation. The simulation CT scan was fused with prostate MRI to assist in contouring. The initial clinical target volume (CTV1) was defined as the prostate capsule, proximal or entire seminal vesicles, and if applicable pelvic lymph nodes. A boost clinical target volume (CTV2) was also contoured and included the prostate capsule alone. Adjustments were made, taking into consideration the prostate biopsy and MRI results. The CTVs were typically expanded 6 mm superiorly and inferiorly and 4 mm in all other dimensions to create a pair of planning target volumes (PTV1 and PTV2). In addition, a PTV_Eval structure was created with lateral-only expansions of 5 mm, which was used to drive dose coverage under robustness evaluation. Organs at risk (OAR), including the rectum, bladder, penile bulb, femoral heads, and small bowel, were contoured using established contouring guidelines as demonstrated in **Figure 1A** [16].

**Figure 1.**
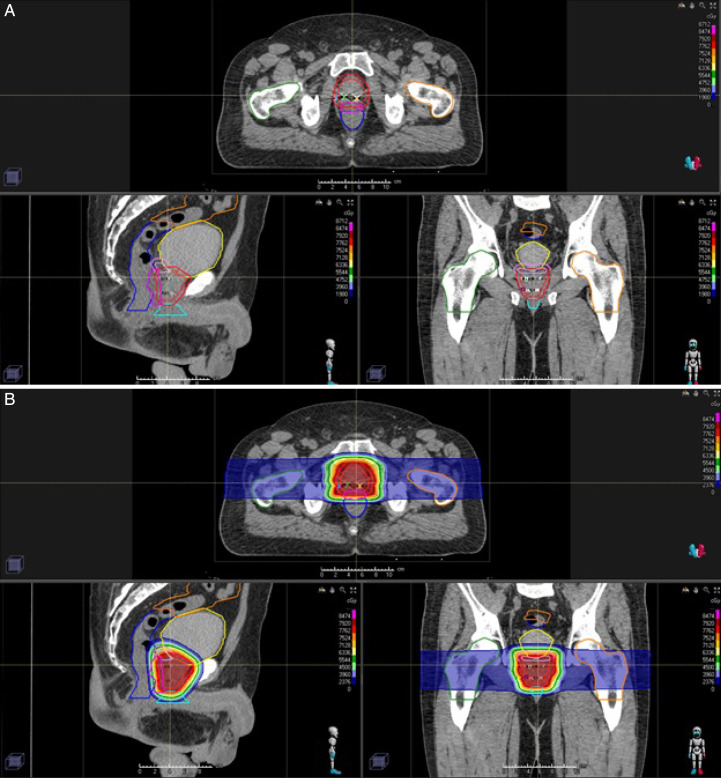
(A) Plan for a patient diagnosed with favorable intermediate risk adenocarcinoma of the prostate. Hydrogel spacer demonstrated by the pink contour creating artificial displacement between anterior aspect of the rectum and posterior aspect of the prostate. (B) Color wash dose distribution of treatment to a total dose of 7920 cGy (RBE) in 44 fractions (initial field including seminal vesicles to a total dose of 4500 cGy [RBE] in 25 fractions) using opposed lateral proton beams. (C) Color wash dose distribution of single lateral plan.

**Figure 1.**
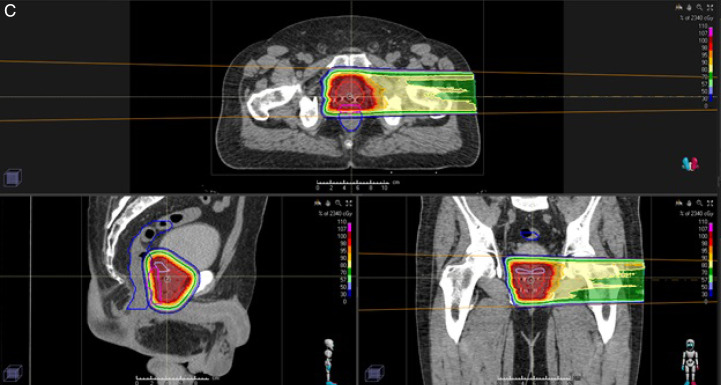
Continued.

### Treatment Planning and Delivery

Dose calculations and planning optimization were performed on the simulation CT scan. Proton plans were generated using RayStation (version 6-9A, RaySearch Laboratories, Stockholm, Sweden). Opposed lateral beam angles were created to optimize target volume coverage and minimize OAR radiation exposure, as demonstrated in **Figure 1B** and **1C**. Robustness evaluation for 6 directional positional shifts of 5 mm and radiation certainty of ±3.5% was used to ensure coverage of CTVs. The majority of cases were planned, optimized, and delivered using 1 lateral field per day to assist in patient and center day-to-day logistical ease. Single field optimization was used for all PBT plans. Of note, in certain situations, particularly with unfavorable seminal vesicle anatomy, cases were planned using SFO but treated with 2 beams daily. As such, each portion of the seminal vesicles did not require highly weighted spots from the contralateral beam owing to the contribution from the ipsilateral beam allowing for some additional shaping around the rectum while still using SFO planning. Moreover, each patient was treated via a 2-stage scheme; ie, to the prostate and seminal vesicles to initial dose (4500 cGy [RBE]) followed by a boost to the prostate only. Thus, unfavorable seminal vesicle anatomy did not contribute to excess high dose to the rectum. All plans were optimized with a Monte Carlo dose calculation algorithm. Apertures were created using the Adaptive Aperture multileaf collimator system (Mevion Medical Systems, Littleton, Massachusetts). Planning overrides were used for rectal gas and artifact created by fiducial markers. Of note, solid gold markers are generally exclusion criteria for protons because of the shadowing defect, thus Visicol markers were used in the present study. Each marker was contoured individually, and any CT artifact was overridden to match prostate density. Subsequently, robust evaluation ensured no shadowing occurred in any of the positional shift scenarios, and if concern arose, the patient was treated using 2 beams per day. All patients were treated with standard fractionation to an initial dose of 4500 to 5040 cGyRBE followed by a cone down to a final prescription dose of 7020 to 8100 cGyRBE. Quality-assurance CT scans were obtained regularly during treatment. Proton beam therapy re-plans were performed, if necessary, to ensure intrinsic anatomical changes during treatment did not significantly alter target coverage or OAR dose constraints. Patients were set up daily using orthogonal kV imaging with gross setup to bony anatomy and final adjustment based on fiducial alignment.

### Follow-up

Patients were evaluated weekly for on-treatment visits during their radiation course, at which time acute side effects were identified. Following treatment completion, patients were seen every 3 months for the first year and subsequently at 6-month intervals thereafter. Posttreatment evaluations included serial PSA levels and clinical examination. In addition, patients were followed by their primary urologist following completion of treatment. Acute and late toxicity were reported using the Common Terminology Criteria for Adverse Events (CTCAE, version 5.0; US National Cancer Institute, Bethesda, Maryland). Acute toxicity was defined as toxicity occurring during radiation treatment and within 90 days of treatment completion. Toxicity was reported and reviewed by the treating radiation oncologist. Patient-reported sexual function and urologic toxicities were also assessed using the American Urological Association (AUA) Symptom Index and Sexual Health Inventory for Men (SHIM) scores at baseline and at each follow-up for the majority of patients. Follow-up was calculated from the date of treatment completion. Biochemical failure was calculated using the Phoenix definition.

## Results

### Patient and Tumor Characteristics

From July 2018 to April 2020, a total of 33 patients were treated with a median follow-up of 18 months. Patient, tumor, and treatment characteristics are delineated in Table 1. The median age of all patients was 75 years, with 85% of patients having an Eastern Cooperative Oncology Group (ECOG) performance status of 0. The most common prostate grade group was 2. The percentage of patients with low-, intermediate-, and high-risk localized prostate cancer was 6%, 45%, and 49%, respectively. Overall, pretreatment urologic function was acceptable, with a median pretreatment AUA of 8, with the majority of patients (79%) having a score less than 15 prior to treatment. Median prostate size was 60.5 cm^3^. Pretreatment SHIM scores were indicative of severe baseline erectile dysfunction throughout our cohort, with a median pretreatment score of 5, likely reflective of the older median patient age. Prior to PBT, 5 patients were treated with alpha-blockers, 2 with alpha-reductase inhibitors, and 1 patient had undergone a transurethral resection of the prostate (TURP). Androgen-deprivation therapy was recommended to 27 patients given their diagnosis risk grouping, although 5 patients who were eligible ultimately declined due to quality-of-life concerns. Of those who received ADT, 6 patients received short-term ADT, and 16 patients received long-term ADT.

**Table 1.** Patient, tumor, and treatment characteristics.

	**Patient No.**	**Percentage**
**Age, y**		
≤70	22	67
>70	11	33
**ECOG scale**		
0	28	85
1	5	15
**PSA, ng/mL**		
<10	16	49
10–20	13	39
>20	4	12
**AJCC 8th edition T-stage**		
T1	22	67
T2	9	27
T3–T4	2	6
**Grade group**		
1	2	6
2	13	40
3	4	12
4	11	33
5	3	9
**Perineural invasion**		
Yes	6	18
No	27	82
**Risk group**		
Low	2	6
Favorable intermediate	4	12
Unfavorable intermediate	11	33
High	13	40
Very high	3	9
**Pretreatment AUA**		
<15	26	79
≥15	6	18
**Pretreatment SHIM**		
<15	16	48
≥15	13	39
**Pretreatment urologic function**		
Alpha blocker	5	15
Alpha-reductase blocker	2	6
TURP	1	3
**ADT**		
Short-term	6	18
Long-term	16	49
**Anticoagulation use**		
Yes	3	9
No	30	91

Abbreviations: ECOG, Eastern Cooperative Oncology Group; PSA, prostate-specific antigen; AJCC, American Joint Committee on Cancer; AUA, American Urological Association; SHIM, Sexual Health Inventory for Men; TURP, transurethral resection of the prostate; ADT, androgen-deprivation therapy.

### Treatment and Dosimetric Characteristics

The majority of patients (76%) were treated with conventional fractionation to a total dose of 7920 cGyRBE or higher. At the time of treatment, there were limited data to support hypofractionated schedules using PBT given radiobiological concerns. A small number of patients were treated to a lower dose because of anatomical variations, primarily excess small bowel in the treatment field precluding delivery of a higher radiation dose. Patients treated to this lower dose reflected a concern related to RBE uncertainties and their effect on bowel as well as a manifestation of our conservative approach early in the opening of our center. Nearly all cases were treated with 2 parallel opposed proton fields except for 2 patients who received pelvic lymph node radiation to 4500 cGy using intensity-modulated radiation therapy followed by boost to the prostate and seminal vesicles using parallel opposed proton fields. Initial institutional practice was to treat with bilateral parallel opposed fields daily (n = 8), which was eventually converted to unilateral daily treatments (n = 25) for logistical ease. Proton dosimetric characteristics are listed in Table 2.

**Table 2.** Dosimetric parameters.

**Dose**	**Patient No.**	**Percentage**
**Radiation, RBE**		
<79.2	8	24
≥79.2	25	76
**Fields per day**		
1	25	76
2	8	24
**Dosimetric parameter**	**Median**	**Mean (range**)
**Target coverage, %**		
CTV1 V100%	100	100 (100–00)
PTV1 V100%	100	99.9 (98.8–100)
CTV2 V100%	100	100 (99.0–100)
PTV2 V100%	98.7	98.5 (92.1–100)
**Rectum, %**		
V75Gy	0.7	1.2 (0.0–8.1)
V70Gy	2.1	2.7 (0.0–12.1)
V65Gy	4.1	4.8 (0.0–16.4)
V60Gy	6	6.7 (0.1–20.8)
V40Gy	19.4	20.8 (2.0–40.8)
**Bladder, %**		
V80Gy	6.9	6.8 (2.1–17.3)
V75Gy	14	13.3 (4.3–25.7)
V70Gy	18	18.1 (5.9–32.0)
V65Gy	23.4	21.8 (7.4–38.7)
V45Gy	36.6	34.7 (6.5–64.5)
**Femoral heads, %**		
Right V35Gy	0.8	1.4 (0.0–7.0)
Left V35Gy	1.6	3.3 (0.0–18.5)
**Penile bulb, Gy**		
Mean	40.2	39.3 (19.5–53.7)
**Small bowel, Gy**		
Dmax	12.3	22.7 (0.3–63.3)

**Figure 2.**
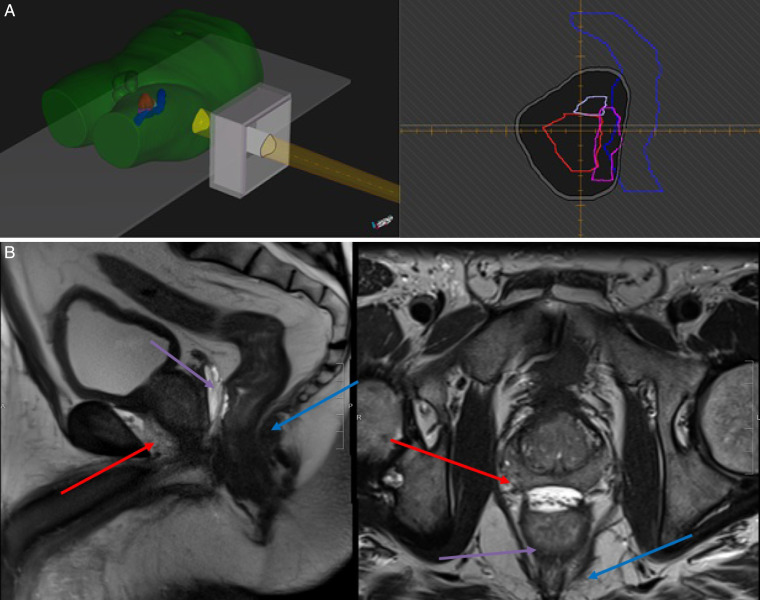
(A) (left) In-room view of single left lateral proton beam field with associated aperture projection. (right) Beam's eye view of aperture projection demonstrating hydrogel spacer displacement, which allows for sparing of rectum and mitigation of radiation dose to this organ at risk. (B) A T2-weighted MRI of the prostate in the coronal (left) and axial (right) planes demonstrating the separation of the prostate (red arrow) and rectum (blue arrow) provided by insertion of a hydrogel spacer (purple arrow).

Cohort median target coverage was excellent, with PBS–PBT achieving 100% coverage to the CTV1 and PTV1 with boost coverage of 100% to the CTV2 and 99% to the PTV2. Cohort median bladder volumetric dose parameters including V80 Gy (RBE), V75 Gy (RBE), V70 Gy (RBE), and V65 Gy (RBE) were 7%, 14%, 18%, and 23%, respectively. These results met our institutional dose objective criteria but are nominally higher than other contemporary dosimetric reports owing to our use of Monte Carlo–based planning [14, 15]. In addition, hydrogel rectal spacing achieved low cohort median rectal dose parameters including V75 Gy (RBE), V70Gy (RBE), V65 Gy (RBE), and V60 Gy (RBE), which were 1%, 2%, 4%, and 6%, respectively. The organ separation achieved by hydrogel spacers is demonstrated in **Figure 2A**, which shows both an in-room beam's eye view of this anterior posterior separation, ideal for lateral proton beams, and **Figure 2B**, which demonstrates the organ separation provided by the hydrogel spacer as viewed on a T2-weighted MRI. Similarly, excellent bilateral femoral head dosimetry was achieved with a cohort median V35 Gy (RBE) less than 2%. Finally, cohort median mean penile bulb dose was 40 Gy (RBE) and cohort median small bowel dose maximum was 12 Gy (RBE).

### Acute Toxicity

High-grade acute toxicities were not observed. As expected, the most common domain of toxicity was GU, with 17 patients experiencing at least 1 grade 2 toxicity. The most common low-grade GU side effects of any grade included frequency (n = 33), urgency (n = 23), and urinary retention (n = 19). Gastrointestinal (GI) toxicity was uncommon, with no patients developing acute grade 2 or higher GI toxicity. The most common rectal toxicity was diarrhea (n = 9). A majority of patients (88%) experienced grade 1 fatigue during treatment. Unique to PBT, many patients experienced lateral pelvic skin hyperpigmentation and erythema (n = 14) during the course of therapy; however, none of these cases progressed to overt moist desquamation. Table 3 demonstrates the details of the aforementioned acute toxicity.

**Table 3.** Acute toxicity (CTCAE, version 5.0).

	**Grade 1**	**Grade 2**	**Grade 3**	**Grade 4**	**Grade 5**
**Genitourinary**					
Dysuria	7	—	—	—	—
Hematuria	2	2	0	0	0
Urinary frequency	17	16		—	—
Urinary incontinence	3	0	0	—	—
Urinary urgency	12	11	—	—	—
Urinary tract infection	0	3	0	0	0
Bladder spasms	0	7	0	—	
Urinary retention	15	4	0	0	0
**Gastrointestinal**					
Proctitis	1	0	0	0	0
Diarrhea	9	0	0	0	0
Fecal incontinence	0	0	0	—	—
Rectal hemorrhage	0	0	0	0	0
Fatigue	29	1	0	—	—
Skin hyperpigmentation	14	0	—	—	—
Insomnia	7	0	0	—	—
Erectile dysfunction	5	4	0	—	—
Anxiety	9	0	0	0	—

Note: Dash indicates not applicable.

**Table 4.** Late toxicity (CTCAE, version 5.0).

	**Grade 1**	**Grade 2**	**Grade 3**	**Grade 4**	**Grade 5**
**Genitourinary**					
Dysuria	4	—	—	—	—
Hematuria	2	1	0	0	0
Urinary frequency	9	9	—	—	—
Urinary incontinence	1	1	0	—	—
Urinary urgency	7	2	—	—	—
Urinary tract obstruction	1	2	0	0	0
Urinary tract pain	1	1	0	—	—
Bladder spasms	0	3	0	—	—
**Gastrointestinal**					
Proctitis	2	0	1	0	0
Diarrhea	1	0	0	0	0
Rectal hemorrhage	1	1	0	0	0
Fatigue	5	0	0	—	—
Skin hyperpigmentation	1	0	—	—	—
Insomnia	1	0	0	—	—
Erectile dysfunction	4	10	0	—	—

Note: Dash indicates not applicable.

### Late Toxicity

Late toxicity was analyzed with a median of 18 months of follow-up. Overall, severe toxicity was rare, with only 1 reported grade 3 GI toxicity event. The most common GI toxicity of any grade was proctitis (n = 3). The solitary severe GI toxicity was categorized as grade 3 radiation proctitis leading to intermittent rectal bleeding, straining, and diarrhea, which began 8.7 months following treatment completion. This led to repeat colonoscopies, which confirmed radiation proctitis at which time argon plasma coagulation was performed. Despite advanced proton planning, spacer placement, and optimal dosimetry, this patient still developed rectal toxicity, which in part could be explained by range and RBE uncertainties. It is important to note, this patient had a longstanding history of cardiovascular disease and was treated prior to radiation therapy with ticagrelor 60 mg by mouth twice daily (Manufacturer, City, State) as well as aspirin. These medications likely contributed to an increased risk of gastrointestinal toxicity. The remaining late GI toxicities were categorized as low grade.

There were no grade 3 or higher GU toxicities identified, and the most common grade 2 toxicity was urinary frequency (n = 9) requiring initiation of tamsulosin (Manufacturer, City, State). A clear resolution of acute GU toxicity was observed over time, and this may be a manifestation of the good pretreatment urinary function of our patient population. Late grade 2 erectile dysfunction was fairly frequent and manifested in a total of 10 patients, which is reflective of the underlying poor erectile function of our cohort evident from pretreatment SHIM scores. Finally, acute fatigue and skin hyperpigmentation demonstrated a resolution with time.

### Early Oncologic Outcomes

With a median follow-up of 18 months, the mean and median PSA nadir were 0.73 and 0.40 ng/mL, respectively. One patient initially diagnosed with high-risk prostate cancer developed a Phoenix definition biochemical failure at 21 months and was subsequently diagnosed with bone metastases on restaging scans and initiated on enzalutamide. Of note, 1 patient was immediately lost to follow-up.

## Discussion

Here we report the clinical outcomes of patients with localized prostate cancer treated with definitive PBS–PBT using Monte Carlo–based planning, fiducial-based image guidance, and hydrogel rectal spacing without the use of endorectal balloons. Our early experience demonstrates excellent dosimetry using active scanning proton therapy with Monte Carlo planning resulting in low dose–volume parameters for the bladder and, particularly, the rectum. In our study population, we demonstrate low rates of acute GI toxicity, with no patients reporting grade 2 or higher acute GI toxicity, comparing favorably with other evaluations of proton therapy early toxicity [19, 20]. Similarly, no patient experienced acute grade 3 or higher GU toxicity. We did identify 1 case of late grade 3 GI toxicity requiring argon plasma coagulation approximately 9 months after treatment completion in one of the 3 patients on anti-coagulation prior to, during, and fater radiotherapy.

Historically, proton centers primarily used passive scatter delivery systems, which required creation of patient-specific compensators, apertures, and yielded less conformal proton dose distributions. Pencil-beam scanning systems have optimized proximal target dose distribution and allowed intensity-modulated proton therapy to be employed. However, improvements in dose conformality may come at a cost of greater sensitivity to anatomical changes, including variations in bladder and bowel filling leading to inter- and intrafractional motion. However, observations indicate that these variations may not have a clinically significant impact on treatment delivery [21, 22]. Moreover, the use of hydrogel rectal spacers may mitigate any clinically significant changes caused by organ perturbations, from a rectal standpoint, owing to prostate stabilization and augmentation of distance between the prostate and rectum [23]. Our experience with spacers sans rectal balloons demonstrates it to be a safe and feasible way to deliver PBS–PBT.

Hydrogel spacers are polyethylene glycol-based gels, which create a geometrical expansion of the potential space between the posterior aspect of the prostate and the anterior aspect of the rectum. As a result, considerable separation between the prostate and rectum is achieved resulting in rectal dosimetric improvements across nearly all dose–volume parameters [6, 9]. These dosimetric improvements have translated into particularly low rates of clinical GI toxicity and excellent patient-reported quality of life for intensity-modulated radiation treatments [6, 7, 11, 24–26]. One proton dosimetric study also demonstrated excellent radiation dose reductions to the rectum and theoretical reductions in normal tissue complication probability (NTCP) and predicted toxicity with the use of hydrogel spacers for prostate cancer [27]. Critical to proton therapy, hydrogel spacers also provide improved anatomical reproducibility, which has been shown to be comparable with the daily use of endorectal balloons without the patient discomfort and inconvenience of a balloon [8, 12, 28].

Dinh and colleagues [29] published a powerful investigation that retrospectively analyzed GI toxicity in patients treated with both passive scatter and PBS proton therapy with either rectal balloon immobilization or rectal hydrogel spacer placement. In this analysis, significantly lower GI toxicity was observed with the use of rectal hydrogel spacers. Similarly, the largest study with the longest follow-up to date for PBS–PBT with spacer placement *combined* with rectal balloon immobilization included 51 patients who demonstrated reductions in rectal dosing with hydrogel placement [30]. However, with 9.5 months of follow-up, there did not appear to be a difference between low-grade rectal toxicity for those with and without spacer placement. In the present study, at 18 months of follow-up, we demonstrate minimal GI toxicity with the use of hydrogel spacers sans endorectal balloons with exclusively PBS–PBT, distinct from the aforementioned publications.

In our study population, overall rates of GU toxicity are consistent with previously published studies, despite overall higher doses delivered to the bladder, which are likely a manifestation of the use of the Monte Carlo dose calculation algorithm. Several studies have demonstrated that pencil-beam dose calculation algorithms, when compared with Monte Carlo, consistently underestimate the delivered dose, whereupon renormalization with Monte Carlo results in higher doses delivered to adjacent normal structures [31, 32]. Monte Carlo–based planning creates more accurate dosimetry, particularly in situations of tissue heterogeneity, adaptive planning, and target motion. It is likely that Monte Carlo–based proton planning will become the rule rather than the exception in the future given advantages in dosimetric evaluation. Generally, the risk of acute grade 3 or higher GU toxicities is under 3% following proton therapy treatment [3, 19, 20, 33–35]. Our grade 3 or higher GU toxicity rate was 0% with early follow-up and limited numbers, which is in line with other published results.

Limitations of this study include the nonrandomized and retrospective nature of the analysis, which may promote patient-selection biases. Additionally, the follow-up period is relatively short for the disease site and is only capable of evaluating early toxicities and oncologic outcomes. However, despite these limitations, we feel that the present study provides a unique addition to the literature in examining the outcomes for patients with prostate cancer treated with PBS–PBT in conjunction with hydrogel rectal spacers sans endorectal balloon immobilization.

The future of proton therapy for localized prostate cancer will trend toward moderate and extreme hypofractionation, similar to the movement seen in its x-ray–based counterparts. Grewal et al [36] recently published the 4-year experience of a prospective phase II trial exploring acute and late toxicity for moderately hypofractionated proton therapy delivered to a total dose of 70 Gy (RBE) in 28 fractions. Overall, excellent rates of biochemical relapse-free survival were reported and only 1 late grade 3 or higher toxicity was observed. Similar impressive outcomes have been reported by other single institutions for moderately hypofractionated proton therapy in the treatment of localized prostate cancer [37–39]. The results of extreme proton hypofractionation, in contrast, are limited, and mixed results have been observed. The PCG GU002 demonstrated similar clinical results between 38 Gy (RBE) in 5 fractions versus 79.2 Gy (RBE) in 44 fractions [40]. Likewise, a study published by Kubeš et al [41], demonstrated promising early clinical outcomes with low rates of relapse and acute toxicity. Alternatively, a phase II trial by Ha et al [42], demonstrated potentially inferior biochemical failure free survival in patients treated with extreme hypofractionation (35 CGE in 5 fractions over 2.5 or 5 weeks). Additional prospective trials and longer-term follow-up will be required to determine if these hypofractionated schemas are as efficacious and safe as their x-ray counterparts.

## Conclusions

Early clinical outcomes of patients treated for localized prostate cancer using modern PBS–PBT with Monte Carlo planning, hydrogel rectal spacing sans endorectal balloons, and fiducial tracking demonstrates minimal acute and late toxicity with good early oncologic outcomes. Future research and longer follow-up will be required to monitor long term cancer outcomes and late toxicity.
